# Clinical significance of a novel inflammatory-nutritional index in glaucoma severity evaluation

**DOI:** 10.17305/bb.2025.12374

**Published:** 2025-05-27

**Authors:** Xiao Xiao, Yanping Gao, Gao Zhang, Zuo Wang, An Li, Donghua Liu, Jing Fu, Wenbo Xiu, Chang Lu, Jinxia Wang, Xiong Zhu, Yang Chen, Lingling Chen, Bolin Deng, Ping Shuai, Chong He, Fang Lu

**Affiliations:** 1 Clinical Immunology Translational Medicine Key Laboratory of Sichuan Province, Sichuan Provincial People’s Hospital, University of Electronic Science and Technology of China, Chengdu, China; 2 Core Laboratory, Sichuan Provincial People’s Hospital, School of Medicine, University of Electronic Science and Technology of China, Chengdu, China; 3 Department of Clinical Laboratory, Sichuan Clinical Research Center for Cancer, Sichuan Cancer Hospital & Institute, Sichuan Cancer Center, Affiliated Cancer Hospital of University of Electronic Science and Technology of China, Chengdu, China; 4 Health Management Center, Sichuan Provincial People’s Hospital, University of Electronic Science and Technology of China, Chengdu, China; 5 Department of Electronic Communication and Technology, Shenzhen Institute of Information Technology, Shenzhen, China; 6 Department of Prenatal Diagnosis, Chengdu Women’s and Children’s Central Hospital, School of Medicine, University of Electronic Science and Technology of China, Chengdu, China; 7 Department of Immunology, West China School of Basic Medical Sciences; & Forensic Medicine, Sichuan University, Chengdu, China; 8 Department of Ophthalmology, Sichuan Provincial People’s Hospital, University of Electronic Science and Technology of China, Chengdu, China; 9 Medico-Engineering Cooperation on Applied Medicine Research Center, University of Electronic Science and Technology of China, Chengdu, China

**Keywords:** Glaucoma, albumin, Alb, lymphocyte rate, biomarker, systemic inflammation

## Abstract

This study investigated the association between glaucoma and serum albumin (Alb), lymphocyte percentage (LYMPH%), and their combined index (LAP ═ LYMPH% × Alb), to evaluate their potential as biomarkers for systemic inflammation and disease progression in glaucoma. We enrolled 161 glaucoma patients and 181 healthy controls. Serum Alb and LYMPH% were measured using standard blood biochemistry and routine tests, and LAP was calculated accordingly. Statistical analyses were performed to compare these markers between groups and assess their correlation with disease severity. Both the median serum Alb level and peripheral blood LYMPH% were significantly lower in the glaucoma group compared to controls (Alb: 43.48 g/L vs 44.63 g/L, *P* < 0.001; LYMPH%: 24.25% vs 29.12%, *P* < 0.001). Correspondingly, LAP levels were also significantly reduced in glaucoma patients (1053 vs 1298, *P* < 0.001). Lower LYMPH% and LAP levels were associated with more severe glaucomatous visual impairment (LAP, healthy controls vs glaucoma: AUC ═ 0.7080, *P* < 0.001, Max Youden ═ 0.3621; early vs severe glaucoma: AUC ═ 0.8061, *P* < 0.001, Max Youden ═ 0.5377). In summary, LAP may serve as a supportive biomarker of systemic inflammation in glaucoma. It demonstrates good accuracy in reflecting glaucoma severity and shows potential for monitoring disease progression.

## Introduction

Glaucoma, a group of diseases characterized by visual dysfunction and optic neuropathy [1], is projected to affect 100 million individuals globally by 2040 [2]. Current challenges in early detection and prevention have driven researchers to explore innovative diagnostic and therapeutic strategies using advanced biotechnological approaches [3, 4]. While animal models have provided valuable insights into the biochemical pathways involved in glaucoma, their findings do not always translate seamlessly to non-invasive clinical applications [5, 6]. Growing evidence suggests that systemic inflammation plays a key role in the etiopathogenesis of glaucoma [7]. As a result, systemic inflammatory markers have emerged as promising tools for disease diagnosis and prognosis [8–10]. Peripheral venous blood analysis, due to its cost-effectiveness, simplicity, and accessibility—even with minimal sample volumes—offers a practical approach for screening and diagnosing high-risk individuals [11, 12]. In our previous study, we found that serum albumin (Alb) and bilirubin—both known for their strong antioxidant and anti-inflammatory properties—were significantly lower in patients with glaucoma compared to healthy controls [13]. Furthermore, both markers showed a negative association with the clinical severity of visual impairment in glaucoma patients. Other research groups have also reported the clinical utility of systemic inflammatory indices in glaucoma management, including blood cell counts (neutrophils and lymphocytes) [14], complement C3 [15], uric acid [16], and platelets [17]. Beyond individual parameters, recent studies have focused on composite markers, such as the neutrophil-to-lymphocyte ratio (NLR) [18], the platelet-to-lymphocyte ratio (PLR) [17], and the lymphocyte-to-monocyte ratio (LMR) [14], as direct indicators of systemic inflammation. These biomarkers have shown considerable promise as early detection tools and for individualized glaucoma screening [19]. In our earlier work, we identified a significant positive correlation between the neutrophil-to-Alb ratio (NAR) and the severity of visual impairment, demonstrating its strong predictive value for glaucoma severity [13]. To further enhance the clinical utility of serum Alb in glaucoma assessment, we developed a novel composite index: the product of lymphocyte percentage (LYMPH%) and serum Alb concentration, which we termed the lymphocyte–Alb product (LAP). We hypothesized that LAP would outperform Alb or lymphocyte levels alone in monitoring disease severity in glaucoma patients. To test this hypothesis, we conducted a single-center retrospective study of glaucoma patients, analyzing LAP levels and their association with disease severity. Our findings support LAP’s potential as a clinically useful biomarker for tracking glaucoma progression. Given its non-invasive nature, cost-effectiveness, and ease of measurement, LAP may aid in assessing disease severity and guiding personalized treatment strategies for glaucoma patients.

## Materials and methods

### Patients

In accordance with the Declaration of Helsinki, we recruited 161 glaucoma patients and 181 healthy controls, all over 18 years of age. Each glaucoma patient underwent their first comprehensive eye exam, which included assessments of corrected distance visual acuity, intraocular pressure (IOP), retinal nerve fiber layer (RNFL) thickness, spectral optical coherence tomography, and static visual field testing. Patients with pre-existing conditions, such as diabetes, chronic renal failure, rheumatism, hypertension, hyperlipidemia, anemia, cancer, or myocardial infarction were excluded. Additionally, individuals taking medications known to affect blood cell counts or serum biochemistry were also excluded. We classified the severity of glaucoma based on visual field results, categorizing cases as mild, moderate, or severe. The visual field was divided into four concentric zones, and each was assigned a score from 0 to 20. A score of 0 indicated no measurable defect, while a score of 20 reflected the presence of at least two depressed points in the nasal area and nine depressions in each hemifield. These scores were used to grade the severity of visual field loss, which was then further quantified using mean deviation (MD) grading. Healthy controls were recruited from examination centers and selected based on the following criteria: daytime IOP below 21 mmHg, RNFL thickness within normal limits, no angle closure, a vertical cup-to-disc ratio (VCDR) less than 0.3 and/or symmetric cupping and/or large optic discs without atrophy, and no visual field defects. Participants were also screened for neurological disorders, which were ruled out.

### Clinical examination

Blood samples were collected from the antecubital (anterior elbow) vein of non-fasting patients. Blood imaging, C-reactive protein (CRP), and erythrocyte sedimentation rate (ESR) analyses were performed immediately after sampling. Peripheral blood was drawn into EDTA-anticoagulated Vacutainer CPT tubes (BD Biosciences), while serum was collected using serum separator tubes (BD Biosciences). All samples were processed within 30 min. Blood cell counts were performed using a Mindray BC-5500 system, and serum Alb levels were measured on an Architect C16000 analyzer (Abbott) in the hospital’s biochemistry laboratory.

### Ethical statement

This case-control study involved subjects recruited from both outpatient and inpatient departments at Sichuan Provincial People’s Hospital, a comprehensive provincial hospital located in Chengdu, China. All participants provided written informed consent for the use of their clinical data. The study was conducted in strict accordance with the Declaration of Helsinki and received full approval from the Institutional Review Board for Clinical Research at Sichuan Provincial People’s Hospital (Approval No: 2024019).

### Statistical analysis

GraphPad Prism (version 9; GraphPad Prism Software, Inc.) was used for statistical analysis. All data were first tested for normality using the Kolmogorov–Smirnov test. The levels of lymphocytes, ALB, and lipid accumulation product were compared between the two groups using a non-parametric rank-sum test. Categorical variables were analyzed using the chi-square test. Data that conformed to a normal distribution were presented as mean ± standard deviation. The unpaired two-tailed Student’s *t*-test was used to compare variables between glaucoma patients and healthy controls, except for age. For data that did not follow a normal distribution, group differences were assessed using the Mann–Whitney test. A *P* value of <0.05 was considered statistically significant. ROC analysis was performed to determine optimal cutoff values for predicting PACG, which were then used to calculate the sensitivity and specificity of LAP probabilities in predicting glaucoma. After confirming the predictive value of LAP as a continuous variable, we evaluated its correlation with LAP classifications.

## Results

### Demographics of the participants

In this study, 161 glaucoma patients were enrolled, including 99 with PACG and 62 with primary open-angle glaucoma (POAG). The control group consisted of 181 individuals with a similar age and sex distribution. Patients were categorized into three groups based on the MD of the visual field and the severity of visual field loss: Early (*n* ═ 34, MD > −6 dB), Moderate (*n* ═ 63, MD between −6 and −12 dB), and Severe (*n* ═ 64, MD ≤ −12 dB). If a patient had glaucoma in both eyes, one eye was randomly selected for data collection. Detailed demographic and descriptive statistics for both glaucoma patients and healthy controls are presented in Table 1.

**Table 1:** Demographics and parameters of glaucoma patients and healthy controls

	**Glaucoma**	**Healthy controls**	***P* value**
*n*	161	181	–
Age (year)	61.4 (±13.1)	70.0 (±9.2)	<0.001
*Gender*			
Female	87	69	0.003
Male	74	112	
PACG/POAG	99/62	-	
*Glaucoma severity*			
Early	34	–	–
Moderate	63	–	–
Severe	64	–	–
LYMPH%	24.25 (±7.098)	29.12 (±7.455)	<0.001
Alb (g/L)	43.48 (±2.867)	44.63 (±2.514)	<0.001
LAP	1053 (±313.5)	1298 (±333.3)	<0.001

All data were expressed as median (IQR) or mean ± SD. Kolmogorov–Smirnov was used to test the normality. Data conforming to the normal distribution were presented as mean ± standard deviation. The unpaired Student’s *t*- (two-tailed) test was used between the glaucoma patients and the healthy control group except for age. If the data did not conform to the normal distribution, the differences between the two groups were analyzed by Mann-Whitney test. Statistically significant, *P* < 0.05. IQR: Quartile range; PACG: Primary angle-closure glaucoma; POAG: Primary open-angle glaucoma; LYMPH%: Lymphocyte percentage; Alb: Albumin; LAP: Lymphocyte rate-albumin index.

### LAP levels are significantly decreased in patients with glaucoma

As reported in Table 1, serum Alb levels were significantly lower in the glaucoma group (43.48 ± 2.867 g/L) compared to the control group (44.63 ± 2.514 g/L, *P* < 0.001), consistent with our previous findings. We also examined the LYMPH%, which showed a reduction in glaucoma patients relative to controls [13]. While prior studies have focused on absolute lymphocyte counts, LYMPH% has not yet been explored in the context of glaucoma. As previously noted, we developed a novel composite marker—LAP—defined as the product of LYMPH% and serum Alb concentration. Notably, LAP was significantly lower in glaucoma patients (1053 ± 313.5) than in controls (1298 ± 333.3, *P* < 0.001). Given that age and gender may influence systemic inflammation, we also conducted a multivariate analysis. The results indicated that LAP remained significantly different between glaucoma patients and healthy individuals (LAP: OR 0.997, 95% CI: 0.996–0.998, *P* < 0.001; Table S1). However, no significant differences in LAP levels were observed between patients with primary angle-closure glaucoma (PACG: 1067 ± 338.3) and POAG (1032 ± 270.7, *P* ═ 0.491; Table S2).

### Associations of LAP with disease severity of patients with glaucoma

**Figure 1.**
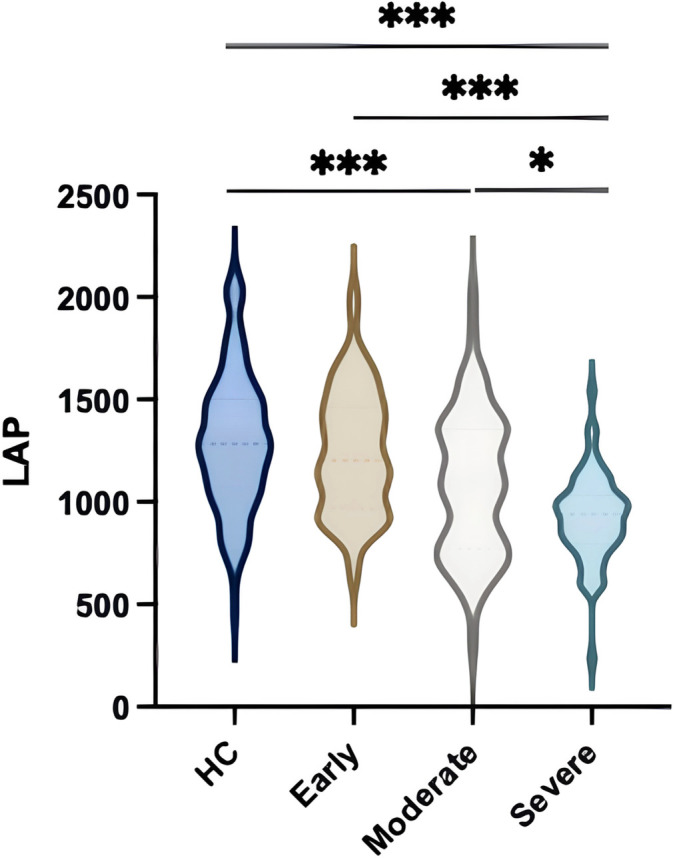
**Relationship between serum albumin, lymphocyte rate, LAP and severity of disease.** Different groups of glaucoma patients stratified by disease severity (A) LYMPH%, (B) Alb, and (C) LAP levels. *P < 0.05, **P < 0.01, ***P < 0.001. LYMPH%: Lymphocyte percentage; Alb: Albumin; LAP: Lymphocyte rate-albumin index.

Subsequently, we shifted our focus to evaluating the clinical relevance of LAP in glaucoma. To this end, we categorized patients based on the severity of their visual impairment. As shown in Figure 1, LAP values demonstrated a clear downward trend from the Early to the Severe group. This decline was statistically significant when comparing both the Early and Moderate groups to the Severe group. These findings suggest a potential association between LAP levels and glaucoma progression, with LAP decreasing as the disease advances. This relationship highlights LAP’s potential utility as a biomarker for monitoring and assessing glaucoma severity.

### The correlation between LAP and glaucoma analyzed by ROC curve

**Figure 2.**
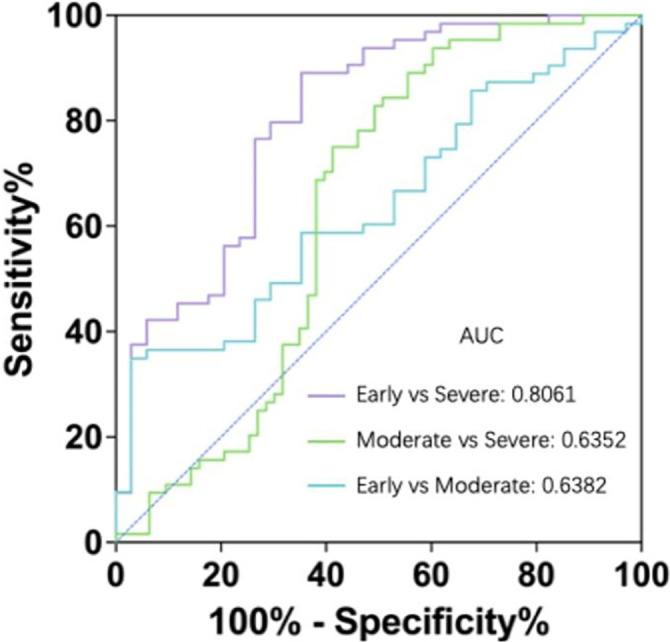
**Receiver operating characteristic curve analysis of LAP in glaucoma patients with different severity.** The severity of glaucoma was determined based on the mean deviation of visual field and the degree of visual field defect. AUC, area under the curve. Early vs Severe, *P* < 0.001, Moderate vs Severe, *P* ═ 0.009, Early vs Moderate, *P* ═ 0.025. LAP: Lymphocyte rate-albumin index.

Having established that LAP levels are significantly lower in glaucoma patients compared to controls, we proceeded with a more comprehensive evaluation of LAP’s diagnostic utility using ROC curve analysis. As presented in Table 2, LAP demonstrated respectable discriminatory power for distinguishing glaucoma from control subjects (AUC ═ 0.7080, *P* < 0.001), outperforming ALB (AUC ═ 0.6151) and LYMPH% (AUC ═ 0.6894) (Figure 2). Building on our prior findings—which identified serum total bilirubin, indirect bilirubin, the NAR, the NTBR, and the NIBR as potential discriminators between glaucoma and controlgroups—we conducted a comparative analysis of these indices against LAP. In this context, LAP again showed the highest AUC (LAP: 0.7080; NAR: 0.6673; NTBR: 0.6338; NIBR: 0.6566), underscoring its superior performance. Further, to evaluate LAP’s potential in assessing disease severity, we applied ROC analysis to LAP values stratified by clinical severity. Notably, LAP demonstrated the greatest capacity to differentiate severe from early-stage glaucoma (AUC ═ 0.8061, *P* < 0.001; Max Youden Index ═ 0.5377), surpassing all previously reported markers, including ALB, LYMPH%, total and indirect bilirubin, NAR, NTBR, and NIBR. These findings collectively suggest that LAP is not only a useful marker for identifying glaucoma but also holds promise as a tool for stratifying disease severity and monitoring progression (Table 3).

**Table 2:** The discriminative abilities of variables between patients with glaucoma and healthy controls

	**GL vs HC**				
	**AUC**	***P***	**Max. Youden**	**95% CI**	**Cut-off** **(sensitivity %, specificity %)**
ALB	0.6151	<0.001	0.2028	0.5556–0.6746	42.85 (76.8%, 43.48%)
LYMPH%	0.6894	<0.001	0.3214	0.6333–0.7455	26.15 (66.3%, 65.84%)
LAP	0.7080	<0.001	0.3676	0.6531–0.7630	1141 (69.06%, 67.7%)

Receiver operating characteristics (ROC) curve analysis. Youden index: sensitivity + specificity − 1. Statistically significant, *P* < 0.05. AUC: Area under the ROC curve; Max. Youden: The Youden index maximum; LYMPH%: Lymphocyte percentage; Alb: Albumin; LAP: Lymphocyte rate-albumin index.

**Table 3:** The discriminative abilities of variables in patients with glaucoma, stratified according to disease severity

	**Early vs Moderate**					**Early vs Severe**					**Moderate vs Severe**				
	**AUC**	***P***	**Max. Youden**	**95% CI**	**Cut-off** **(sensitivity %, specificity %)**	**AUC**	***P***	**Max. Youden**	**95% CI**	**Cut-off** **(sensitivity %, specificity %)**	**AUC**	***P***	**Max. Youden**	**95% CI**	**Cut-off** **(sensitivity %, specificity %)**
Alb	0.5532	0.397	0.1761	0.4311–0.6735	45.15 (79.37%, 38.24%)	0.5409	0.507	0.1480	0.4212–0.6606	45.15 (76.56%, 38.24%)	0.5166	0.747	0.0741	0.4158–0.6175	44.75 (32.81%, 74.6%)
LYMPH%	0.6256	0.042	0.2246	0.5132 –0.7379	18.65 (25.4%, 97.06%)	0.7813	<0.001	0.4770	0.6803–0.8822	26.05 (85.94%, 61.76%)	0.6360	0.008	0.3207	0.5360–0.7361	24.3 (79.69%, 52.38%)
LAP	0.6382	0.025	0.3198	0.5271 –0.7492	864.4 (34.92%, 97.06%)	0.8061	<0.001	0.5377	0.7119 –0.9002	1130 (89.06%, 64.71%)	0.6352	0.009	0.3373	0.5337–0.7366	1019 (75.00%, 58.73%)

Youden index: Sensitivity + Specificity − 1. The severity of glaucoma was determined based on the mean deviation of visual field and the degree of visual field defect. Statistically significant, *P* < 0.05. PACG: Primary angle-closure glaucoma; POAG: Primary open-angle glaucoma; LYMPH%: Lymphocyte percentage; Alb: Albumin; LAP: Lymphocyte rate-albumin index.

## Discussion

The pursuit of non-invasive methods for monitoring visual deterioration in glaucoma remains a significant clinical challenge, as current techniques are inadequate for definitively assessing disease progression [19]. There is an urgent need for innovative, reliable tools that can track functional visual changes without invasive procedures. Addressing this gap would represent a major advancement in glaucoma management and patient care. Building on our previous finding of an inverse correlation between serum Alb levels and the clinical severity of visual impairment in glaucoma patients [13], we propose a novel composite biomarker: the LYMPH% multiplied by serum Alb concentration (LAP). This serological index integrates hematological (LYMPH%) and biochemical (Alb) parameters to enhance the clinical utility of serum Alb in glaucoma assessment. Our study yielded two key findings: first, glaucoma patients exhibit significantly lower LAP levels compared to healthy controls; second, LAP correlates strongly with disease severity, supporting its potential as a clinical evaluation tool. The development of LAP as a composite index offers several advantages for assessing glaucoma severity. By combining LYMPH% and serum Alb, LAP provides a more comprehensive and nuanced view of the disease. LYMPH% reflects immune activity, which is increasingly recognized as a factor in glaucoma pathogenesis. Serum Alb, meanwhile, serves as a surrogate marker for physiological functions, such as vascular integrity [20] and nutritional status [21]. Integrating these parameters into a single index offers a more holistic representation of the underlying disease mechanisms. Incorporating LAP into routine clinical practice could provide clinicians with valuable insights into disease progression, supporting more informed treatment decisions. Its non-invasive nature enhances patient comfort and suitability for long-term monitoring. Overall, LAP represents a promising tool for personalized and targeted glaucoma management.

Numerous research findings have highlighted the significant involvement of peripheral immunity in glaucoma. Specifically, we observed that the circulating CD4+ T cell response is enhanced in parallel with the progression of visual damage in glaucoma patients. Our data demonstrate a positive correlation between T cell activation and disease severity. Additionally, we identified an inverse relationship between serum Alb levels and the clinical severity of visual impairment. The redox state of vitreous Alb has been proposed as a biomarker for assessing the oxidative environment in patients with POAG. Similarly, elevated levels of ischemia-modified Alb have been documented in patients with PACG, underscoring its potential as a marker of oxidative stress. In the present study, we aimed to enhance the utility of Alb and lymphocytes by combining these two parameters through multiplication of serum Alb and the peripheral blood LYMPH%. We termed this new index the LYMPH%–Alb product (LAP) and investigated whether it could more effectively monitor clinical activity and nerve injury progression in glaucoma patients compared to serum Alb or LYMPH% alone. ROC analysis of LAP data from 161 patients and 181 healthy controls yielded an AUC of 0.7080, with a maximum cutoff value of 1141. At this threshold, sensitivity and specificity were 69.06% and 67.7%, respectively, indicating diagnostic value. When comparing LAP across disease stages, the diagnostic efficacy improved: early- vs severe-stage analysis produced an AUC of 0.8061, with a cutoff of 1130, achieving a sensitivity of 89.06% and a specificity of 64.71%. These results are relatively satisfactory and suggest that multi-marker analyses incorporating correlated indicators may help identify more effective biomarkers. Beyond its established role as a nutritional marker, serum Alb serves as a vital antioxidant in plasma, continually combating oxidative stress [22]. Although the pathogenesis of glaucoma remains complex and largely enigmatic [23], growing evidence points to the importance of oxidative/antioxidative imbalance in both human and animal studies. Alb functions like a “tramp steamer” in circulation, scavenging various molecules, including transition metals (e.g., copper and iron) and polyunsaturated fatty acids [24–26]. When oxidized, these molecules can generate reactive oxygen species (ROS). Thanks to its ligand-binding capabilities, Alb exerts multiple antioxidant effects [27]. While earlier views dismissed impairments in Alb’s antioxidant capacity as biologically insignificant, emerging evidence suggests otherwise. Compromised Alb function has been associated with conditions, such as diabetes and chronic kidney disease [28]. Moreover, oxidized Alb has been proposed as a marker of oxidative stress in neurodegenerative diseases like Alzheimer’s and Parkinson’s [29, 30]. Recent research has further explored the redox state of Alb in glaucoma, reinforcing its potential as a biomarker. For instance, the redox state of vitreous Alb has been linked to the oxidative milieu in POAG [31], while elevated ischemia-modified Alb levels in PACG highlight its relevance in assessing oxidative stress [32]. We speculate that the upregulation of oxidative stress responses in glaucoma leads to increased consumption of Alb, which may contribute to reduced serum Alb levels [33]. Additionally, some reports suggest that proteinuria in glaucoma patients may further drive down serum Alb concentrations [34].

Alterations in the levels of CRP and interleukin-6 (IL-6) in glaucoma, along with their associations with the disease, warrant further investigation. In patients with PACG, serum levels of IL-6, IL-8, and high-sensitivity CRP (hs-CRP) were significantly elevated compared to those in the normal control group. Additionally, IL-6 levels were positively correlated with the degree of visual field defect and the VCDR. In patients with POAG, aqueous humor IL-6 levels were significantly and positively correlated with intraocular pressure. These findings suggest that changes in CRP and IL-6 levels may be closely related to glaucoma pathogenesis, including inflammatory responses, optic nerve damage, and vascular abnormalities. Detecting these biomarkers could provide valuable references for clinical diagnosis and treatment. However, we are currently seeking more accessible and cost-effective biomarkers. To this end, we have explored the combined use of lymphocyte rate and serum Alb as potential indicators and are actively investigating other meaningful biomarkers. Future research may focus on integrated analyses to support auxiliary diagnosis in clinical settings—an approach that is relatively simple and economical, though not without challenges. We will continue to address these issues in future in-depth studies. Several study limitations must be acknowledged. First, lymphocyte rate and serum Alb are general indicators of systemic inflammation or nutritional status and are not specific to glaucoma. Moreover, the current sample size is relatively small. It is necessary to validate the efficacy of these markers in a larger cohort and to design multicenter, cross-regional studies to assess the generalizability of LAP as a marker of disease severity. Second, the cross-sectional and retrospective nature of this study limits our ability to draw conclusions about the causal relationship between LAP levels and glaucoma severity. To better explore the temporal relationship, future studies should include long-term, regular follow-up with large sample sizes. In addition, future research should account for potential confounding factors—such as comorbidities and medications—to improve the accuracy and robustness of LAP as a clinical tool. Finally, many studies in the field of non-invasive biomarkers for optic nerve injury in glaucoma have integrated hematological and biochemical parameters—such as the NLR, NAR, PLR, and NIBR—to identify early indicators of nerve damage. These existing markers typically rely on combinations of two parameters. Our goal is to develop more comprehensive, multi-level composite biomarkers by integrating multiple hematological and biochemical indicators to support early diagnosis and clinical decision-making through in-depth research.

## Conclusion

In conclusion, our study successfully developed and evaluated the clinical utility of a novel lymphocyte- and Alb-based index, LAP, for assessing disease severity in glaucoma patients. The reduced LAP levels observed in these patients, along with the strong correlation between LAP and clinical severity, underscore its potential as a valuable tool in glaucoma management. LAP’s comprehensive assessment capabilities surpass those of many previously studied markers, offering a more accurate evaluation of disease status. While further research is needed to confirm its reliability in routine clinical practice, this non-invasive biomarker holds promise for transforming glaucoma care and enhancing patient outcomes.

## Supplemental data

**Table S1:** Comparison of the glaucoma group and the control after multivariate logistic regression analysis

**Characteristics**	**Glaucoma**	**Control**	**OR (95% CI)**	***P* value**
Age, M (SD)	61.4 (±13.1)	70.0 (±9.2)	0.950(0.929–0.972)	<0.001
Sex (Male), *n*(%)	74 (46.0%)	112 (61.9)	2.058 (1.266–3.347)	0.004
LAP	1053 (±313.5)	1298 (±333.3)	0.997 (0.996–0.998)	<0.001

*n*(%) indicates the number of constituents (*n*) and the composition ratio (%). LAP: Lymphocyte rate-albumin index.

**Table S2:** Demographics and parameters of PACG and POAG

	**PACG**	**POAG**	***P* value**
*n*	99	62	–
Age, years	64 (55–70)	58.5 (53.75–72.25)	<0.001
*Glaucoma severity*			
Early	20	14	
Moderate	39	24	
Severe	40	24	
Alb (g/L)	43.20 (41.90–45.20) *↓*	44.00 (41.98–45.20)	<0.001
LYMPH%	23.40 (19.50–30.10) *↑*	22.85 (19.48–28.35)	<0.001
LAP	1067 (±338.3)	1032 (±270.7)	0.491

All data were expressed as median (IQR) or mean ± SD. Kolmogorov–Smirnov was used to test the normality. Mann–Whitney test was used for differences in all indicators except LAP in PACG and POAG patients. Statistically significant, *P* < 0.05. PACG: Primary angle-closure glaucoma; POAG: Primary open-angle glaucoma; LYMPH%: Lymphocyte percentage; Alb: Albumin; LAP: Lymphocyte rate-albumin index.

## Data Availability

The datasets generated and/or analysed during the current study are not publicly available. Due to privacy protection and data security concerns, the data cannot be shared. However, upon reasonable request, the data may be made available from the corresponding author.
